# Supplementary material for: Transforming Motivation for Exercise in a Safe and Kind Environment—A Qualitative Study of Experiences among Individuals with Type 2 Diabetes
Source: Int J Environ Res Public Health. 2022 May 17;19(10):6091. doi: 10.3390/ijerph19106091 (PMC9141646; doi:10.3390/ijerph19106091)
Supplement: Supplementary file 1 [file ijerph-19-06091-s001.zip › ijerph-1675947-supplementary.pdf]

Supplementary Material

Table S1. Initial themes and sub-themes.

Themes	Sub-Themes
Experiences with exercise	Experiences from before
	Exercise intensity
	Taking initiatives/signs of empowerment
	Role of the physiotherapist
	Feeling safe
Sustaining physical activity	Barriers
	Home exercises
	Planning
Who we are	Having diabetes
	Special needs
	Focusing on feet
	Women's class
Motivation	Being healthy
	Feeling good
	Social atmosphere
Class structure	Time and day
Satisfaction	Transportation
